# Sex differences in formal recommendation of assets for health (social prescribing) in Aragon

**DOI:** 10.1186/s12889-024-19497-4

**Published:** 2024-07-25

**Authors:** Marina Pola-Garcia, Carmen Belen Benede Azagra, Natalia Enriquez Martin, Maria Luz Lou Alcaine, Elena Melus-Palazon, Fatima Mendez-Lopez, Angel Gasch-Gallen

**Affiliations:** 1 Centro de Salud Almozara, Sector Zaragoza II, Servicio Aragonés de Salud, Zaragoza, Spain; 2 Grupo GIIS011, Instituto de Investigación Sanitaria de Aragón (IISA), Zaragoza, Spain; 3 Centro de Salud Canal Imperial, Sector Zaragoza II, Servicio Aragonés de Salud, Zaragoza, Spain; 4 Red de Investigación en Cronicidad, Atención Primaria y Prevención y Promoción de la Salud (RICAPPS) RD21/0016/005, Instituto de Salud Carlos III, Madrid, Spain; 5 Grupo Aragonés de Investigación en Atención Primaria B21_23R, Gobierno de Aragón, Zaragoza, Spain; 6 Unidad de Calidad y Seguridad, Servicio Aragonés de Salud, Departamento de Sanidad, Gobierno de Aragón, Zaragoza, Spain; 7 Dirección General de Asistencia Sanitaria y Planificación, Departamento de Sanidad, Gobierno de Aragón, Zaragoza, Spain; 8 Centro de Salud Amparo Poch, Sector Zaragoza I, Servicio Aragonés de Salud, Zaragoza, Spain; 9 Departamento de Medicina, Psiquiatría y Dermatología, Universidad de Zaragoza, Zaragoza, Spain; 10 Departamento de Fisiatría y Enfermería, Facultad de Ciencias de la Salud, Universidad de Zaragoza, Zaragoza, Spain; 11 Grupo GIIS094, Instituto de Investigación Sanitaria de Aragón (IISA), Zaragoza, Spain; 12 Zaragoza, Andador de Aragüés del Puerto 50015 Spain

**Keywords:** Sex, Gender role, Health Promotion, Social Prescribing, Primary Health Care

## Abstract

**Background:**

In primary health care, social prescribing is an important tool which is gaining popularity. It is being studied significantly, however there is not enough evidence about different related issues. The aim of this study is to analyse the differences by sex in the application of a social prescription protocol in Primary Care.

**Methods:**

This is a cross-sectional study carried out with data from the Electronic Health Record between September 2018 and March 2021. Descriptive, bivariate and multivariate analyses of data from 2,109 records of Social Prescription protocol in primary health care centers located in Aragón in northern Spain (Europe) were performed using Jamovi Statistics software (version 2.3.28). The comparisons by sex were carried out using a Mann-Whitney U or chi-squared test to analyse differences.

**Results:**

The protocol was used correctly 1,482 times, where it was applied more in females (74.8% female vs. 25.2% male). The median age in females was higher than males (female 72 vs. males 70; *p* = 0.003). There were significant differences by sex in several aspects to strengthen with the social prescribing, physical, emotional and relational skills. Most females and males regularly attended the recommended asset and there were significant differences in the group that never attended. Mean satisfaction was statistically different, with 4.74 points out of 5 for females and 4.86/5 for males (*p* = 0.010). It can be observed that older females in rural areas (OR = 34.15), whose social prescription acts on Emotional Skills and Relational and Social Skills (OR = 6.10–8.23), with good prior self-care and greater participant satisfaction (OR = 8.96), have greater chance of improving their health.

**Conclusions:**

Some results showed sex differences in the use and outcomes of formal asset recommendation. However, further research is needed to assess the relationship between social prescription, sex and gender and their implications.

## Background

Positive Health focuses on aspects that allow individuals, families and communities to increase control and improve their health. This way of looking at health is related to the theory of Salutogenesis and Asset Based Health [1].

In 2007, Morgan A. y Ziglio E. [2] defined the concept “Health Asset” as “any factor (or resource), which enhances the ability of individuals, groups, communities, populations, social systems, and institutions to maintain and sustain health and well-being and to help to reduce health inequities. Assets can operate at the individual, group, community, and population level as protective (or promoting) factors to buffer against life’s stresses.”

In primary health care (PHC), social prescribing (SP) is an important tool to deal with some of the needs of people who come to consultations and support them in having better control of their health [2, 3]. This is defined as the referral of people who attend Primary Care consultations to different local non-clinical services (assets). [3]

Kimberlee established four levels of social prescribing based on the degree of structure and coordination between health services and health assets that participated in prescribing [4]. Some variations of this classification exist, for example, that which differentiates between non-formal (Level 1 of Kimberlee) and formal (Levels 2, 3 and 4 of Kimberlee) social prescription [5].

Social Prescribing is an increasingly current topic and a practice which is becoming more and more formal in community attention in PHC teams in Spain and other countries [6].

There are some documents and guides which includes SP as an essential activity in PHC, and they propose some implementation models [7–11].

Aragón, a region of Spain (Europe), has been working on the issue for a long time and it has a guide about the process [12]. This guide describes the necessary phases for implementing formal schemes of SP [12]. For its application, a Social Prescription Protocol is included in the Electronic Health Record and a searcher of health assets helps to visualise the existing assets and choose the most adequate [13].

Social prescribing aims to improve people’s health and reduce health inequalities. It is therefore often targeted at vulnerable individuals or groups [2, 14]. However, there is not enough evidence to suggest who can benefit from it [14, 15], and very few studies show differences with it by sex [16].

There is some evidence which shows general differences in health and illness depending on the sex and gender [17].

When assessing the differences between the sexes, it is necessary to consider the conditioning of being a female and her role in the family, social functioning and work expectations. These simultaneous processes worsen and limit the health status of females along with the influence of biological, psychological and social factors that condition them. These social determinants influence the frequency, vulnerability, or severity of health problems. How symptoms are perceived, and the access and use of health services are affected too [18–20]. This issue is hierarchical and produces inequalities [17].

The lack of knowledge and correct adaptation of health attention depending on these differences can influence in the continuity of these inequities.

Considering that social prescription attempts to contribute to the reduction of inequalities, the present study aims to describe and analyse differences by sex in the use and impact of a social prescription protocol in primary health care in Aragón (Spain - (Europe)).The second objective of this study is to analyse the association between improvement in health perceived by the prescribing health professional and some factors related to the patient such as sociodemographic data, satisfaction perceived by the patient, assistance and areas to strengthen.

## Methods

### Study design

This research project is a cross-sectional study carried out with data from the Electronic Health Record (EHR) recorded between September 2018 and March 2021 in all Health Primary Care centres in Aragón, in northern Spain (Europe).

### Contextual framework

The study was conducted within the framework of primary health care in Aragon, a region of Spain (Europe). In 2021, Aragon had a population of 1.331.938 inhabitants Here, the population is distributed among big urban areas (in the biggest city of the region, Zaragoza, 675,301 inhabitants live), medium sized areas and rural areas. Municipalities with a population of less than 10,000 inhabitants are considered rural areas. With 22.5% of the population over 65, Aragon has an ageing population. [21, 22] In Aragon, the public health system serves almost the entire population, with each individual assigned to a health center in the area where he or she resides. Around 70–85% of the population prefers public primary health care over private. Aragon public primary health care is structured into eight health sectors organised into 123 Basic Healthcare Areas. Spanish primary health care comprises a multidisciplinary team [23, 24].

### Social prescription protocol

Within the framework of primary health care in Aragon, the Social Prescription protocol has been included in the EHR since its incorporation in September 2018. Furthermore, for its registration and opening in the EHR, social prescription must always be linked to a diagnostic.

When starting the SP, the aspects to be strengthened (physical, social, mental and emotional skills, among others), the reason for the prescription and the proposed asset must be recorded. This protocol also includes the need for an assessment by a social worker. During follow-up, some information about assintance, satisfaction, and improvement is reported. Likewise, the protocol is linked to a health asset search engine to view the existing assets in the area and choose the most appropriate [13].

### Subjects and sample size

The sample consisted of all patients with an open EHR held by health centres in the region of Aragon (Spain) with a social prescription protocol opened. Accordingly, the inclusion criteria were: (i) people of all ages, (ii) registered a social prescription protocol. The exclusion criterion was to minimize inconsistencies in the data. The data studied were the product of the clinical practice of Primary Care professionals in Aragon. Due to the universal nature of the healthcare system and the absence of other PHC providers, the data obtained in the study is considered representative of practically 100% of the studied population.

Each registry entry to the protocol includes hosting or monitoring records. In total, there were 2,109 records in the EHR. The extracted data were reviewed and all recording errors were removed (*n* = 199).

**Fig. 1:**
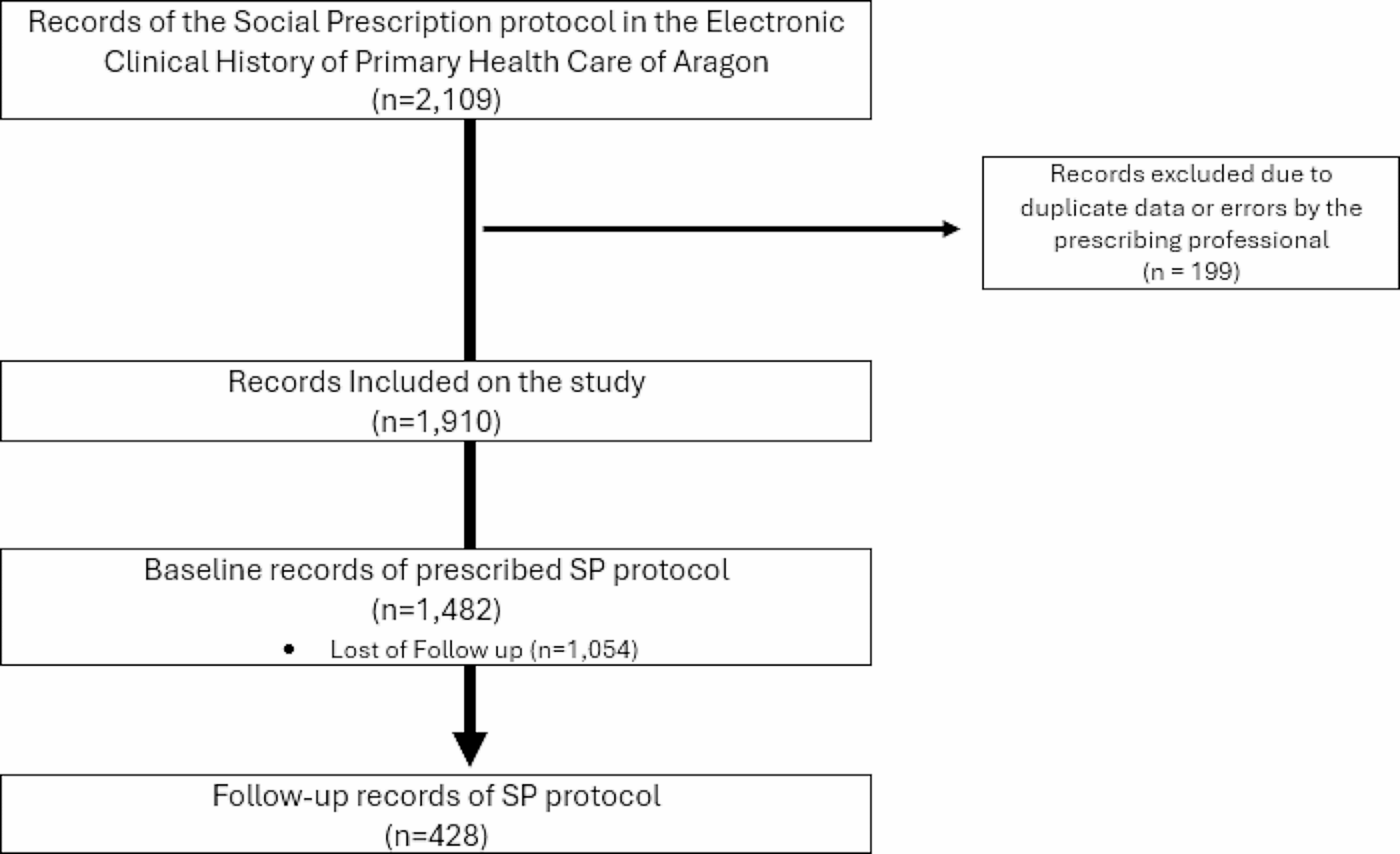
Flowchart of the source data collection method. Adapted from Pola-Garcia M, Domínguez García M, Gasch-Gallén Á, Lou Alcaine ML, Enríquez Martín N, Benedé Azagra CB. Implementación de un protocolo de recomendación formal de activos para la salud en los equipos de atención primaria aragoneses. Aten Primaria. 2022;54 [25]

Finally, 1482 initial records of the SP protocol were included in the study, of which only 428 were complete follow-up records (Fig. 1).

### Variables

All the data were collected from the EHR.

Sociodemographic data: information on sex (females or males), age (both the age in its value and grouped into different ranges [up to 60 years, from 61 to 80 or 81 and older]) and residence (rural or urban) were collected.

Social prescription protocol data: information on the health problems according to the International Classification of Primary Care (ICPC) [26] to which the recommendation was associated; the areas to be strengthened with the prescription (self-care, physical skills, social skills, mental skills, emotional skills, or the category “other” that refers to any aspect not included in the previous options); the frequency of attendance at the prescribed health asset (frequently, occasionally (when attendance is less than 80%) or never); the improvement perceived by the health professional (Likert scale 0 to 5, 5 being the maximum improvement) and the patient satisfaction (Likert scale 0 to 5, 5 being the maximum satisfaction) were collected.

### Statistical analysis

The Kolmogorov-Smirnov test was used to evaluate the normality of the scores derived from all quantitative variables using statistical methods.

In our case, all quantitative variables showed a non-normal distribution, so non-parametric statistical methods were used. A descriptive analysis (frequencies (n) and percentages (%) for categorical variables;, median (Me) and Interquartile range (IQR) for continuous variables) was performed to characterize the sample.

Second, bivariate analyses were conducted using the chi-square test for qualitative variables and the Mann-Whitney U for quantitative variables, aiming to compare the different variables between females and males.

Multivariate ordinal logistic regression models were constructed to evaluate the association between the improvement in health perceived by the prescribing health professional and some factors related to the patient such as sociodemographic data, satisfaction perceived by the patient, assistance and areas to strengthen. Adjusted ordinal OR (aOR) and 95% CI were calculated. The proportional odds assumption was evaluated using the likelihood ratio test.

Due to the number of losses to follow-up, the data have been analyzed separately; On the one hand, the baseline characteristics have been analyzed with the 1,482 initial records, and on the other hand, the analysis of the follow-ups and the characteristics related to them has been carried out, performing simple imputation and eliminating those records that did not contemplate follow-up, only for these analyses. For the logistic regression, only the data of the 482 people who had follow-up were used. A statistical significance level of *p* < 0,05 was fixed. All analyzes were performed using Jamovi Statistics software (version 2.3.28).

## Results

The SP protocol had 1,482 initial records during the study period. According to the patient’s sex, 1,108 (74.8%) of the social prescription was done to females and 374 (25.2%) to males.

The median age in females was 72 (IQR 19), and in males was 70 (IQR 18), showing a statistically significant difference (Mann-Whitney U 185,765; *p* = 0.003). Statistically significant differences were found between females and males according to age group. As can be seen in Table 1, the highest proportion of people were between 61 and 80 years old for both sexes. However, differences were found, with the highest proportion being in the male group at younger ages (up to 60 years) and in the female group at older ages (61–80 years). There were no differences regarding the urban or rural origin of the participants.

**Table 1:** General description of the study population and differences by sex

		Total *N* (%)	Females *N* (%)	Males *N* (%)	χ² (df); *p*
**Age**	**Until the age of 60**	385(26)	267(24.1)	118(31.6)	10.871(2); 0.004
	**From 61 to 80**	758(51.1)	570(51.4)	188(50.3)	
	**81 and more years**	339(22.9)	271(24.5)	68(18.2)	
**Zone**	**Urban**	959(65.1)	719(65.3)	240(64.3)	0.113(1); 0.737
	**Rural**	515(34.9)	382 (34.7)	133(35.7)	

Note: χ²: chi-square test; df difference; p: *p*-value

Regarding the ICPC (Table 2), the diagnoses associated with Chapter Z of Social Problems were the most prominent in females, and the diagnoses associated with Chapter P of Psychological Problems were the most prominent in males. Significant differences by sex were found in some chapters. Specifically, in psychological issues and the grouping of seldom used chapters Both are higher in men.

**Table 2:** Description of the chapters of the ICPC associated with SP and differences by sex

International Classification of Primary Care	Total (*n* = 1,428)	Females (*n* = 1,108)	Males (*n* = 374)	χ² (df); *p*
**A. General and unspecified**,** n (%)**	49(3.31)	33(2.98)	16(4.28)	1.48(1);0.224
**K. Circulatory**,** n (%)**	67(4.52)	49(4.42)	18(4.81)	0.09(1); 0.753
**L. Musculoskeletal**,** n (%)**	142(9.68)	114(10.29)	28(7.49)	2.53(1); 0.111
**N Neurological**,** n (%)**	27(1.82)	15(1.35)	12(3.21)	5.37(1); 0.020
**P. Psychological**,** n (%)**	335(22.6)	228(20.6)	107(28.6)	10.3(1); 0.001
**T. Endocrine**,** metabolic and nutritional**,** n (%)**	132(8.9)	101(9.1)	31(8.3)	0.23(1); 0.627
**Z. Social problems**,** n (%)**	365(24.6)	285(25.7)	80(21.4)	2.83(1); 0.093
**Preventive Activities**,** n (%)**	336(22.7)	264(23.8)	72(19.3)	3.34(1); 0.068
Others*,** n (%)**	32(2.16)	21(1.90)	11(2.94)	1.44(1); 0.223

Note: χ²: chi-square test; df difference; p: *p*-value; * includes ICPC chapters whose total number is less than 10 records (B Blood, hematopoietic organs, lymphatics, spleen, D Digestive, F Ocular, R Respiratory, S Skin, U Urology, W Pregnancy, childbirth, family planning, X Female genital system and breast)

In relation to the sphere or spheres to be strengthened (Table 3), the aspect that was encouraged the most in both sexes were physical skills and the least were cognitive ones. In men, social prescribing associated with non-protocol areas was even higher than the last one. Statistically significant differences were observed in physical (*p* = 0.020), emotional (*p* = 0.003) and relational skills (*p* = 0.007) between men and women, with the highest proportion observed in the female group. Self-care and other spheres were the issues in which the proportion of males was higher. Table 3.

The median number of spheres to be enhanced in both groups 2 (IQR 1), without significant differences between both groups (Mann-Whitney U 218630.00; p 0.089).

**Table 3:** Description of the areas to be strengthened associated with SP and differences by sex

Spheres to be strengthened	Total (*n* = 1,428)	Females (*n* = 1,108)	Males (*n* = 374)	X^2^ (df); *p*
**Physical Skills (Yes)**,** n (%)**	884(59.6)	680(61.4)	204(54.5)	5.41(1);0.020
**Self-Care (Yes)**,** n (%)**	574(38.7)	416(37.5)	158(42.2)	2.60(1);0.107
**Cognitive Skills (Yes)**,** n (%)**	475(32.1)	367(33.1)	108(28.9)	2.31(1);0.128
**Emotional Skills (Yes)**,** n (%)**	604(40.8)	476(43.0)	128(34.2)	8.84(1);0.003
**Relational and Social Skills (Yes)**,** n (%)**	558(37.7)	439(39.6)	119(31.8)	7.25(1);0.007
**Others**	424(28.6)	309(27.9)	115(30.7)	1.12(1);0.290

Note: χ²: chi-square test; df difference; p: *p*-value

Regarding the follow-up (Table 4), most people regularly attended the recommended asset. However, significant differences between both groups who never attended the asset were found. The improvement recorded by professionals was high and coincident for both groups [Female mean 4.74; median 5 (IQR 0) vs. male mean 4.86; median 5 (IQR 0) Mann-Whitney U 9507; *p* = 0.010].

The satisfaction was also high in the two groups, but there was a significant difference in superiority in males. [Female mean 4.27; median 5 (IQR 1) vs. male mean 4.27; median 4 (IQR 1) Mann-Whitney U 6604; *p* = 0.634].

**Table 4:** Description of the attendance, satisfaction and improvement and differences by sex

	Total (*n* = 482)	Females (*n* = 336)	Males (*n* = 92)	χ² (df); *p*
**Attendance, ** *n* ** (%)**				
**Never**	10(2.4)	2(0.6)	8(8.8)	20.3(1); <0.001
**Occasionally**	28(6.7)	23(7.1)	5(5.5)	0.227(1); 0.599
**Regularly**	379(90.9)	301(92.3)	78(85.7)	3.76(1); 0.052
**Satisfaction**,** n (%) (Likert Scale)**				
**1**	3 (0.8)	2 (0.7)	1 (1.3)	13.43(4); 0.009
**2**	1 (0.2)	0 (0.0)	1 (1.3)	
**3**	9 (2.5)	9 (3.1)	0 (0.0)	
**4**	52 (14.4)	48 (16.9)	4 (5.1)	
**5**	295 (81.9)	224 (79.1)	71 (92.2)	
**Improvement**,** n (%) (Likert Scale)**				
**1**	4 (1.3)	3 (1.2)	1 (1.7)	10.379(4); 0.035
**2**	8 (2.6)	7 (2.8)	1 (1.7)	
**3**	40 (13.2)	37 (15.1)	3 (5.3)	
**4**	100 (33.2)	72 (29.3)	28 (50.0)	
**5**	149 (49.5)	126 (51.4)	23 (41.0)	

Note: χ²: chi-square test; df difference; p: *p*-value

To test whether the prescribing health professional perceives that health improvement is associated with all the variables and analyze their differences between female and male, we built two different ordinal logistic regression models (Table 5). Social prescription that acts on Emotional Skills and Relational and Social Skills, with good prior self-care and greater satisfaction of the participants, has a greater probability of improving their health status, these factors representing up to 49% of the probability ( Table 5). However, in the second model we evaluated how sex influences these variables, by studying interactions of variables included in the ordinal logistic regression model. It is observed how health improvement is mediated by sex, specifically, older females in rural areas, whose social prescription acts on Emotional Skills and Relational and Social Skills, with good prior self-care and greater participant satisfaction, have greater chance of improving their health. health status, these factors representing up to 54% of the probability (Table 5).

**Table 5:** Multivariate Ordinal Logistic Regression Model of the improvement recorded by professionals by sex

Model		B	SE	OR 95%CI	*p*-value	R2 Nagelkerke
	Zone (Rural)	-0.030	0.426	0.971 [0.421–2.238]	0.945	0.497
	Sex (male)	0.142	0.327	1.152 [0.603–2.171]	0.665	
	Physical Skills	0.576	0.631	1.177[0.516–6.134	0.361	
	**Self-Care**	**-1.118**	**0.4985**	**0.327 [0.123–0.869]**	**0.025**	
	Cognitive Skills	-0.968	0.5219	0.380[0.137–1.057]	0.064	
	**Emotional Skills**	**1.786**	**0.5322**	**5.966 [2.102–16.932]**	**0.001**	
All variables	**Relational and Social Skills**	**1.572**	**0.4729**	**4.816[1.906–12.168]**	**0.001**	
	Others spheres	-1.050	0.6097	0.350[0.106–1.156]	0.085	
	Age	0.012	0.0224	1.012[0.969–1.058]	0.593	
	Attendance	0.987	0.5673	2.684[0.883–8.161]	0.082	
	**Satisfaction**	**1.912**	**0.3378**	**6.764[3.488–13.115]**	**0.000**	
Interaction Between sex and the different variables*	**Sex (Female) x Self-Care**	**-1.195**	**0.5966**	**0.303 [0.094–0.975]**	**0.045**	
	**Sex (Female) x Emotional Skills**	**2.109**	**0.6146**	**8.236 [2.469–27.473]**	**0.001**	0.543
	**Sex (Female) x Relational and Social Skills**	**1.810**	**0.5912**	**6.108 [1.917–19.459]**	**0.002**	
	**Sex (Female) x Satisfaction**	**2.193**	**0.3954**	**8.964 [4.130-19.457]**	**0.000**	
	**Sex (Female) x Age x Zone (Rural)**	**3.531**	**1.6694**	**34.151 [1.295-900.329]**	**0.034**	

B: unstandardized coefficient; SE: standard error; OR: Odds Ratio; CI: confident interval; *All possible interactions with the sex variable available in the model were included; Only those statistically significant for the model are shown in the table.

## Discussion

This study analysed the use of a social prescription protocol in primary health care in Aragon, as well as the association between the improvement in health perceived by the prescribing health professional and some factors related to the patient such as sociodemographic data, satisfaction perceived by the patient, assistance and areas to strengthen. There are clearly differences related to the sex of the participants in applying a social prescription protocol in the context of a Spanish region, Aragon. It is observed how the protocol was applied more in older females, in whom the protocol was applied more in areas in physical, emotional and relational skills. Furthermore, it can be observed that older females in rural areas, whose social prescription acts on Emotional Skills and Relational and Social Skills, with good prior self-care and greater participant satisfaction, have greater chance of improving their health.

The difference in the number of recommendations made to females and males during the observed period may be influenced by some well-known factors, such as the higher frequency of women attending primary care consultations (9.6 in women and 7.5 in men consultations per year) [27]. However, sex and gender biases, may also be an influential factor [17, 18]. For example, a recent report from the Spanish Ministry of Equality reveals that women use health services more frequently than men and It relates this to greater longevity and a high rate of comorbidities, as well as to the greater presence of women as caregivers of elderly or sick people [28].

Previous studies point to the need to consider sex differences and the implications of unequal gender power relations as essential to making health promotion interventions more effective [29].

In the study, we identified that females are recommended at an older age than males. A first approximation to this fact could be explained by females’ longer life expectancy, which means that the proportion of females in more advanced stages is more significant than that of men [27]. Still, this fact may also be related to the different activities and daily routines that are more common among both groups. The recommendations focus on the ages at which the work activity has finished. However, women maintain unpaid occupations, such as household chores [30] or informal care [31], so the social prescribing may be postponed. This could imply that only at an older age when they cannot take on such tasks, they access activities that enhance their self-care [32]. And perhaps it also needs to know if older males seek less help.

The differences between females and males in the ICPC and its associated chapters may be related to the fact that the health problems recorded and managed in Primary Care have a different distribution and prevalence by sex [33]. However, to understand and study this subject in depth, it would be necessary to continue researching and perhaps using other existing classifications to check it.

Regarding the spheres to be strengthened using the recommendations, the differences found in physical, emotional, relational and social skills are particularly noteworthy, with more emphasis on females. Concerning physical skills, it is essential to note that women generally get less exercise than men [34]. Perhaps the topic of Emotional Skills is influenced by the management and expression of emotions of women and men, especially at the ages observed in the study [35, 36]. The sphere of relational and social skills is perhaps conditioned by the greater loneliness of women [32].

Although there are no significant differences, self-care is the only area in which the proportion of men is more significant than that of females ; perhaps this is related to traditional masculinity, especially years ago. At that time, self-care was not seen as a need for this group [37], so when men reach certain ages or situations, they may need support to promote it. In addition, this may also be influenced by the fact that health professionals reproduce models of care for men and demands on women in the field of self-care in these groups [38, 39].

The differences in the degree of attendance to the asset can be compared to the differences in adherence to treatment in the groups studied. In general, women seem to take more responsibility for their health and disease situation, and they are more adherent than men [40], as it is also the case for the program’s attendance associated with the recommended asset.

To discuss the differences in satisfaction between the two groups, we have not found related studies. However, regarding the general satisfaction expressed with the public health system, men also value it more highly than women do [27].

The degree of health improvement coincident in both groups differs from another study, which showed that males may have greater benefit from social prescribing than females [16]. In the multivariate ordinal logistic regression, we found different characteristics according to sex that influence the level of improvement. This result may be of interest to identify profiles that can benefit more from social prescribing [15].

### Strengths and limitations

Among the strengths of the research is the opportunity to investigate from the beginning and in parallel to the implementation of social prescription in primary care in the Aragones Primary Care Teams [41]. Moreover, it is one of the first analyses to identify sex differences in social prescribing and to contribute to a more equitable implementation of this tool.

Several limitations must be considered in this study.Firstly, the study has a cross-sectional design, since a single extraction of all PS records was carried out, in a period 2018–2021, this limits the ability to draw causal inferences or determine the direction of causal relationships. Furthermore, another of the limitations of the study and more specifically of how the protocol system is established, is that each entry from the registry to the protocol (included as a new entry, whether it is an initial data or a follow-up), therefore our decrease from 2,109 registrations to 1,428 initials. It would be interesting to be able to do a more detailed follow-up over time, evaluating all areas longitudinally. Future prospective studies with a large sample size are warranted to validate our findings [42].

Secondly, the topic studied, social prescription, is a relatively new tool in primary care, so we still do not know the scope it has and the maximum impact it can have on health. Likewise, since it is a new tool, it may have many registration or structural errors that, through these investigations, are intended to be improved in future versions. Likewise, being a novel protocol does not allow us to find sufficient bibliographic evidence to compare the results obtained with those of other previous studies or other contexts.

Third, it can be observed that there is a greater application of the protocol in female than in male, and that this may influence the results obtained, taking into account a possible selection bias; however, as has already been mentioned in the methodology, the population has been selected through daily practice, being the most representative of our PHC environment. It is true that it is important to highlight that there is a greater use of women to health services, either for their health or for the health of other family members who are under their care. It is important to take this into account, carry out more detailed studies. Finally, sex and gender dimensions are still poorly analysed in studies on population health promotion, and the gender approach is not fully considered in health systems [43].

## Conclusions

Some results showed sex differences in the use and outcomes of formal asset recommendation. However, further research is needed to find out why this occurs and how sex and gender influence the way in which professionals use this tool, its impact on health and whether social prescription contributes to reducing the differences associated with these issues.

This study can be a starting point for assessing the relationship between social prescribing, sex and gender.

## Data Availability

Data analysed during the current study are available on the Intranet of the Aragonese Health Service. Every six months, the Department of Health updates the data from the Social Prescription Protocol of Electronic Health Records in Aragon. The dataset is available from the corresponding author upon reasonable request.Open information about them is offered on https://atencioncomunitaria.aragon.es/2022/06/22/indicadores-asistencia-recomendacion-de-activos-para-la-salud-en-atencion-primaria/ and https://www.aragon.es/-/estadisticas-asistenciales.

## Declarations

### Transparency declaration

The Corresponding Author, on behalf of the other signatories, guarantees the accuracy, transparency and honesty of the data and information contained in the study; no relevant information has been omitted, and all discrepancies between the authors have been adequately resolved and described.

## Abbreviations

PHC: Primary Health Care
SP: Social prescribing
ICPC: International Classification of Primary Care
HER: Electronic Health Record
